# Hooping Cough

**Published:** 1867-02

**Authors:** 


Hooping Cough.—For the past ten years we have used with decided benefit the following prescription suggested by Dr. Alnat, more than a quarter of a century ago:

"Take of carbonate of potossa, a drachm; chochineal, a scruple; boiling water, eight ounces. The dose is a teaspoonful three times a day."

Dr. Alnat contends that there is a "peculiar "acid" generated in the system by this disorder, which is the exciting cause of the spadmotic action of the glottis producing the "whoop," and that to obtain the full effect of the anti-spasmodic and anodyne properties of the cochineal an alkoline solution is essential.

Whether Dr. Alnat's peculiar views are correct in regard to the cause of the most distressing feature of whooping cough we will not discuss; but will state that in quite the majority of cases in which we have prescribed the above remedy, we have had a decided modification of the distressing symptoms.

